# Mr. Cameron on Nitre in Scurvy

**Published:** 1830-04-01

**Authors:** 


### XVIII.

### Nitrate of Potash in Scurvy.

In a letter or report from Mr. Charles Cameron, a naval surgeon, to the Navy Medical Board, (with a sight of which report we have been favoured by one of the medical commissioners) there is given an account of a severe scurvy which broke out among the convicts on board the Ferguson transport, on her passage from Ireland to New South Wales, and which threatened to depopulate the crew, till fortunately it was checked by a solution of nitrate of potash in vinegar, or in a mixture of vinegar and lemon juice. The convicts, 216 in number, were embarked on the coast of Ireland in November 1828, and were then in a low state of health, from deficient nourishment and the depressing passions. Bad weather was experienced on the early part of the voyage, and the convicts suffered greatly from sea-sickness. Their constitutions were thus still farther debilitated, and before the ship crossed the equator, the hospital was full of scorbutic patients, and many others were confined to bed, in a dangerous state. The disease assumed a variety of forms, or rather a number of other complaints became engrafted on the scorbutic diathesis, and were thereby rendered much more formidable. Dysentery, however, was the most prominent feature or form, and affections of the lungs were also very common. Two of the men died of the scorbutic dysentery. When they were preparing to bear away for Rio Janeiro, in order to procure refreshments for the sick, Mr. Cameron tried an old remedy recommended by Patterson many years ago, in his Treatise on Scurvy—namely, nitre. The common stock of this being soon exhausted, a supply was procured from the gun-powder on board. The effects Mr. Cameron describes as almost miraculous—so much so that they abandoned the idea of putting into Rio, and pursued their course to New South Wales, where the convicts landed in unusual good health. The formula and mode of administration will be seen in the following extract.

"But I might add that the most distressing symptoms which my patients complained of, in the early stages, namely, a sense of 'oppression and sinking at the pit of the stomach,' were almost invariably relieved, or totally removed, by a few doses of the medicine. The prisoners themselves were so sensible of its good effects, that I had, for the first time, an opportunity of seeing men crave for medicine, the taste of which was certainly not pleasant; and their complexions were so much improved under its use—changing from a sallow, bloated hue, sometimes approaching to livid, to a clear, healthy colour—that it became matter of surprise to every one.

"The medicine was prepared, and exhibited in the following manner:—Eight ounces of nitre were dissolved in so much vinegar as would make the solution amount to sixty-four ounces. Sometimes equal parts of vinegar and lime-juice were used. A little sugar was generally added to render it more palateable; and about four drops of ol. menth. piperitæ, diffused in a small portion of alcohol, was added to the whole, which rendered it more grateful to the stomach.

"One ounce of this solution was the dose, and was seldom exceeded. From three to eight doses, according to the stage of the disease and the severity of the symptoms, were given at equal intervals during the day—from six o'clock in the morning till eight at night. In general, when the disease was taken early, two or three doses a-day, for a week or ten days, were sufficient; but it appeared to me to be always better to commence with three or four doses, and increase the number gradually—daily if necessary. In the advanced stages a much larger quantity may be taken, and is in fact required, than at the commencement of the disease; but although I have often given the solution to the extent of eight ounces daily, and on one or two occasions exceeded this quantity considerably, and have at the same time watched my patients very closely, I never observed any irritation of the stomach or bowels, or any other inconvenience which could be fairly attributed to it. It is, nevertheless, adviseable to dilute each dose with two or three ounces of water when exhibited. While the constitution is thus being corrected and improved, particular symptoms will require the usual attention.

"It is perhaps proper to notice that, about two years ago, I had occasion to give a solution of nitre in water a fair trial in several bad cases of scurvy, where neither vinegar nor lime-juice could be obtained; and, except that sometimes it did not appear to me to sit so easy on the stomach, with the same good effects on the disease."

